# Foreign

**Published:** 1841-04-17

**Authors:** 


### FOREIGN.

---

### INQUESTS IN MIDDLESEX, HELD BEFORE MR. WAKLEY, M. P.

*Alleged Deaths from Excessive Blood-letting by Venesection.*

*Case I.*—John Shears, a robust, florid-looking man, aged forty-three years, died on the 23d of November, 1840; and his widow complained to the constable of St. Marylebone, that the cause of his death was excessive bleeding from the arm on November 21st, under the treatment of an inexperienced practitioner, who had seen him soon after he had fallen in a fit, in a shop where he was working as a carpenter. The allegation, upon some preliminary inquiry, was found not to be groundless, and the warrant for an inquest to be held on the body, in Great Titchfield street, on the 26th of November, was issued, when

Joseph Sawtell stated that the deceased seemed perfectly well on that morning, until, just after beginning to work, his employer, Mr. Clark, observing that he was proceeding unsatisfactorily about it, told him to "gather up his tools and go home, for he had engaged him as a good workman, whereas he saw that he knew nothing about his business, and was no carpenter at all." On receiving half-a-crown for his wages, deceased said it was not enough; and while Mr. Clark was in the act of tendering him a shilling more, he turned pale and fell, speechless, and insensible for a time, breathing heavily until his neckerchief was loosed. In falling, his head struck the edge of a door, and received a deep wound, three inches long, from which blood enough flowed to soak through a thick mat on the floor. He was picked up, and subsequently carried home, a short distance only, in a chair; the wound open. Before leaving the shop he slightly recovered from the shock, and expressed much agony at some pain, now and then putting his hand to his head.

His wife sent for a "doctor" at once, and in twenty minutes one arrived from a shop which had no name over the door. Expressions of agony (or convulsive motions) were continued by the deceased, who was then abruptly asked "how he was," with the addition that he "must have been drinking again." His pulse was felt, and preparations made for venesection; and tape and a wash-hand basin were supplied for the operation, as the patient sat on the edge of the bed, whence he presently sank to the floor, from the impossibility of holding him up, on account of his writhings, though supported on each side by the policemen who had brought him in, and, sitting on the floor, the bleeding was performed, in the left arm.

Thomas Key, a police-constable, being sworn, testified to the latter of the above facts. His fellow-constable held the basin, which, before the bleeding ceased, was nearly three-fourths full.

The coroner here questioned the witness closely as to the amount, and, in order to enable a better judgment to be formed of the quantity that the witness considered was taken, a basin, similar to the one used, and two quarts, by measure, of porter, were brought into court, and the witness was directed to pour into the former, with exactitude, so much of the fluid as he could swear did not exceed the amount of blood received into the basin. The witness having transferred as much as measured three pints and a half, paused ; not, he said, because that was quite enough, but because he would rather underrate the quantity; and he justified his statement by saying, that when the operation was ended, the blood in the basin washed the end of the thumb of the other policeman, as he gently moved it away. And he added, that, besides that blood, at least half a pint was spilled on the carpet, for it soaked over a surface a yard in diameter. The stream of blood stopped by becoming weak, and deceased, by that time, was excessively feeble. The arm was then tied up by the doctor, and deceased lifted into the bed. Witness was quite certain that no water was in the basin when the bleeding was begun. He stayed with deceased until twenty-five minutes past nine o'clock that morning.

Police-constable 111 E was called in to verify or contradict these statements. He asserted their truth.

The landlady of the house, who was in attendance in the bed-room, as well as the wife, was also sworn. She thought that about three and a fifth pints of blood were taken, besides what was spilled on the floor. The bleeding, she calculated, occupied twenty minutes. The bandage, also, got loose in bed, and some blood, not much, was lost there before its escape was discovered. He had convulsions on Saturday, after which he lay nearly still, occasionally moving his head. On Sunday he was more exhausted and quiet. In the evening he was still feebler, and on Monday afternoon, at ten minutes to one, without having once recovered his sensibility of surrounding objects, he died. He was naturally a high-coloured man in the face; after the bleeding he was very pale, but the colour returned during the convulsive fits.

The coroner here said, that however extraordinary the evidence might appear,—and the witnesses spoke positively, and without hesitation,—he could not allow the inquest to proceed until an examination had been made of the body, to ascertain what effects were to be ascribed to the loss of blood, and what to the extravasation in the brain, which the fall in the shop indicated, especially since he had learned that a fornight before his death he had had an epileptic fit.* He was informed, also, that Mr. Wotton, a surgeon, had subsequently been called in to see the patient; he should, therefore, issue an order to that gentleman to examine the body, and then a history of the symptoms could be given by the same person as had ascertained the proximate cause of death. The practitioner who had bled the patient was at present arraigned. The constable, therefore, had been desired to request his attendance at the delivery of the evidence; and he should, also, together with any medical friend whom he might select, be informed when the examination was to be made, that he might have an opportunity of witnessing the appearances that should be disclosed. The court was then adjourned to

December 1st, when Mr. Wotton, surgeon, of Great Portland street, having heard read the evidence that was given on the 24th November, being sworn, said, that he saw the deceased just after he was bled, on Saturday, the 21st November. He was breathing stertorously, the pupils were dilated, and the face was of a dark red, indicating great congestion; but he observed no symptom which then led him to conjecture that the patient had lost an excessive quantity of blood. In fact, though he had heard that he had been bled, he at once applied leeches at the base of the skull. He did not see how much blood had been taken by the lancet. He ordered the patient calomel, and directed mustard cataplasms to be applied to the feet. In the course of the day, the bandage, having been badly placed on the arm, came off, and some blood escaped in the bed. At his second visit, he found that there had, in the interval, been no evacuation from the bowels, and he therefore prescribed a powerful enema. The man remained unchanged for the better during that day, but on the next morning (Sunday) the pupils indicated improvement, and the face was more natural. No other change, however was manifest. The breathing throughout was stertorous, but he presently sank, and died on the following day.

At the post-mortem examination, nothing unusual presented itself externally excepting that, at the vertex of the head, there was a long, ragged, contused wound of the scalp, the apparent effect of a fall, reaching the pericranium, with contusion around it, three inches in diameter. It was such a wound as would indicate a blow materially affecting the brain. On raising the skull, the membranes all presented an unusually vascular and gorged appearance, especially the arachnoid. On turning back the

* On which occasion he was attended by the same practitioner, but without venesection. It may have been with reference to his suspicions on that occasion, that the practitioner imagined him to be drunk now, at nine, A. M. epileptically. This was the only fit, I ascertained, that his wife had in eighteen years (or longer) known him to have.

skull, and dividing the medulla oblongata, the base of the brain particularly showed the last-mentioned characters. It was more turgid than the membranes. Some coagulated blood was lying under the pons Varolii, where it would naturally have gravitated. The membranes encircling the cord in descending, the theca, were characterised by marks of chronic inflammation, with a thickening of the layers, which, presuming that the man had formerly suffered an epileptic attack, was undoubtedly the result thereof. The skull very thick; no denudation of it under the blow. The right cavity of the thorax contained twenty-four ounces of serum. The lung of that side was not diseased. No effusion on the left side; but the lung adherent there, along its whole extent to the pleura. The pulmonary vessels were not deficient of blood. The heart healthy; somewhat enlarged, but without dilatation; it contained a clot. The liver large and soft, indicating ale-tippling. All the other abdominal and pelvic viscera healthy. The blood-vessels of the body were full, *apparently to abundance.* No kind or degree of anæmia was any where discovered. The muscles were in no respect flaccid or soft, excepting so far as they might have become so *post-mortem.* They in no way indicated an excessive loss of blood during life. There was not bloodlessness in any part of the body. In every respect the frame was normal, as regarded fulness of blood, at the time of death. The young man and a medical friend of his were, both of them, as the coroner had desired, present at the examination, and were now in court. Witness considers that the vascularity of, and the extravasation of blood in the brain, and the concussion probably produced by the fall, were quite sufficient to account for the death.

It was unnecessary to carry the inquiry at the inquest farther. The complaint made against the practitioner that the venesection had produced the death, was in no respect sustained. It was too usual, the coroner observed, to bleed persons who fell, suddenly, insensible; but in the present case an excessive loss of blood, on whatever principle taken,—as the repletion of the body, post-mortem, according to the testimony of the surgeon, proved,—was, considering the extravasation of blood in the brain, the only thing that could give the man a chance of recovery; and he should recommend the jury to return a verdict to the effect, that he died a natural death from that extravasation. The jury at once acted upon this suggestion.

The only question asked by the practitioner at the inquest was this: Was the witness quite sure that the basin did not contain any fluid when the blood began to flow into it? He put it to the policeman after the possibility of that circumstance had been suggested by the court. To this inquiry all of the witnesses replied determinately in the negative.

The witness who fetched "the doctor," and the constable of the court, on being asked what was the name of the practioner, could not furnish it, "because there was no name over the door." His card was afterwards handed in. It was subsequently stated that the shop belonged to a member of the London College of Surgeons, who resided and practised in Poland street, and who had formerly established a branch shop in Gilbert street, since sold or closed; and that the custom was to place a gentleman in the shop to act for the principal as his medical representative, or agent.

*Case* II.—In the following case, the subjoined certificate of the cause of death was forwarded to the registrar of the district, by the surgeon who attended the patient during his illness of three days:—

"London, May 29, 1849.

"I hereby certify that Mr. G. P., of —— street, died on Tuesday morning, at half past two, May 26, of an inflammatory gouty affection of the stomach, ending in mortification, being a natural death."

The coroner was informed of the death, and at the same time told that the patient certainly had been ill with a gouty affection of the stomach, but had, in reality, died from the effects of two inordinate bleedings. An inquest was held on the body, and the following statements elicited:—

May 26, 1840.—G. P., a lieutenant in the army, on half-pay, 56 years old, had been the subject of gout in the left foot and knee for five months, and for the last three days had kept his bed from "a bilious attack." On Sunday, the 24th of May, the patient having excessive and painful hiccough, his wife sent for the medical attendant of the family to see him, and by the direction of that gentleman leeches were applied to the patient's chest, as he had slight pain there when he hiccoughed, and some medicine was sent for him. At eleven o'clock on Monday, the 25th, the patient had vomiting and purging, and the surgeon attended him again, and then bled him in the arm, as he lay in bed, taking away (according to the statement of the widow, at the inquest, which was held in Adam street, on Saturday, the 30th, the patient having died on Tuesday, the 26th,) "three parts of a large wash-hand basin full of blood, desiring Frances Atwood, *the servant*, to untie his arm, and bleed him again, to a pint, at four o'clock, desiring her not to lose her senses at the sight, but to work his fingers about at the bleeding." This the servant was on the point of doing when, as the blood began to flow, the surgeon came in, and took half as much blood in quantity as he had taken in the morning, the patient still *lying* in bed. Neither loss produced syncope. The patient, afraid that all was not right, asked the surgeon if there was "any danger," and was consoled with the assurance that there was "none whatever." The wife repeated the question, and was answered that "there was always danger in a common cold." A mustard poultice to the chest, and afterwards a blister, were applied. The hiccough persisted up to this time, and, in fact, never left him until half an hour before he died. The surgeon ascribed it to "wind on the stomach." He became worse after the bleedings. The surgeon did not repeat his visit. Subsequently to the first bleeding he was weak and restless, and suddenly covered with a clammy cold sweat, and afterwards dropped into a doze. After the second loss of blood, "his eye became dull, and he stared and looked wild, and could not sleep." The bleedings somewhat subdued the hiccough each time. He was pale, and without fever under the attack for which the doctor came; he continually ate fluid arrow-root, and drank toast and water. On Monday morning he sat up on the side of the bed and shaved himself, between ten and eleven o'clock, with his feet resting on a writing-desk, the servant holding a glass before him. He complained of no pain, excepting on hiccoughing, throughout the last two days. He was never at all convulsed. On Monday night he became more composed, and frequently said he was better, but he gradually sank in strength; and on Tuesday morning, having at about two o'clock asked the servant to move his head on to the other pillow, died twenty minutes after. He was quite sensible to the last, and shortly before he ceased to breathe, looked round at the servant, and watched her round the room.

The foregoing evidence being supported, on her examination, by Frances Attwood, the coroner adjourned the inquest to Monday, in order to afford time for an examination of the body to be made.

*June 1st.*—On the reassembling of the jury the following evidence was given by Dr. Marshall Hall:

The examination was made on Saturday afternoon. The body was extremely fat. The mediastinum, the heart, and the intestines, were loaded with it. The brain was healthy. Each cavity of the thorax contained about eight ounces of fluid. The lungs were healthy, but adhered to the pericardium. The right ventricle was filled with a large, firm coagulum of fibrine. Such a coagulum was found in the aorta. The left ventricle was perfectly empty. The valves and the texture of the heart were natural, with the exception of the fatty condition of the heart, and a softness which was the result of decomposition. It was generally adherent to the pericardium. The viscera in the cavity of the abdomen were all healthy. The mucous membrane of the stomach was firm in every part, with a blush of redness at the large curvature, and one spot, of a deep slate colour, near the cardia. The liver was adherent to the diaphragm, and between these two there were several firm bands. The abdominal and intercostal muscles were very florid. Many of the tissues were distended by air, the result of decomposition; by air the cerebral veins were filled, and the submucous tissue of the stomach was raised into numerous bladders, of the size, each, of half a pea. Dr. Hall added, that the period after death was much too late for making a satisfactory examination. The surgeon who had bled the deceased, and a friend, were present at it, and approved the account rendered of it by Dr. Hall, who, in reply to further questions of the court, said, that he found no inflammation of the stomach, nor mortification any where. He considered that the disease of the patient was a violent disorder of the stomach, and that either that, or the fatty condition of the heart, might have produced death, which the bleeding might have accelerated. In fact, he attributed death to those three causes combined.

Some notes, which were taken during an explanation of the surgeon at the inquest, respecting his attendance on the patient, furnish the following outline of his views and proceedings in the treatment:—First called to see him at four or five on Sunday morning; did not attend, but, from description of symptoms, sent an emetic. On a second summons, went, between eleven, A. M., and twelve, on that day. Vomiting a bilious matter; had hiccough, and was very uneasy. Said he had been a long time ill with gout, and had pain at the pit of the stomach. Uneasiness on pressure at that part. Ordered six leeches, and sent a mixture. Monday:—Symptoms aggravated, vomiting severe, still much pain on pressure. Regarding the case as severe inflammation of the stomach, prescribed bleeding, and in a wash-hand basin took twenty ounces. A very heavy man. Said he felt better after it. I said it might be necessary to take a little more, and that if prevented from coming at five in the afternoon, the servant might let a little flow at that time, such as a tea-cup full. In the north it is a common practice to instruct fishermen and others how to remove and take blood from the arm in cases of inflammation. Considering him to be extremely ill, went again at half past four, and found that the bandage had been removed, and the blood already flowing. Took only four ounces on this occasion. Would solemnly swear that. Said to the wife, "There is always danger in these cases, but I hope he will do well." Felt alarmed at his condition at the very first seeing him. He asked, "Is there danger?" Answered, "No," but whispered to Mrs. P., intimating the contrary. Told the son, also, that his father was very ill, and the tears came into his eyes. Never said he had wind in his stomach. He was enormously fat. Such attachments of the heart, liver, &c., are of themselves enough, in certain cases, to produce sudden death. The blood taken was buffy; never saw blood more buffed; had it thrown away after showing its appearances in the room. At the examination of the body after death, saw no inflammation or mortification; no evidence of disease, in fact, so decided as I expected.

I cannot add the charge of the coroner, not having attended the present inquiry; nor, on the same account, can attempt to reconcile apparent contradictions in the evidence; but I have no doubt of the correctness of what I have here arranged, from notes which I preserved at the time on hearing that the inquest had been held. There only remains to be added, that the jury returned a verdict,—"Died from the combined effects of a fatty state of the heart, violent disorder of the stomach, and loss of blood."—*London Lancet.*

---

*Case of Acute Hydrocephalus cured.* By Dr. BIERBAUM, of Dorsten.—A boy, whose head was disproportionately large, had from his birth frequently suffered from indigestion, and the greatest variety of children's diseases, which very much checked the development of his body. In April, 1837, when he had completed his second year, after being chilled in the feet, and taking indigestible food, he was attacked with a gastro-rheumatic fever; and hardly had he got over this, when, in consequence of taking cold again, one of the most destructive of children's diseases came on. The patient could not bear his playthings; became very peevish; complained of violent headach; could not hold up his head, but let it fall from one side to the other, and, when lying down, plunged it deep into the pillow. The temperature of the head was burning hot, while the extremities felt cold; the carotids pulsated strongly; the face was of a corpse-like paleness; the eyes red and intolerant of light; the nose and the external auditory meatus quite dry; the tongue clean and red; thirst great; appetite gone; bowels costive, urine scanty; the pulse frequent and hard. To this were added vomiting, terror, coma vigil, the greatest indifference, seizing the head with the hands, grinding of the teeth, respiration hardly audible or visible, and interrupted by sighs, and repeated exclamations that he was burning or falling. It was equally obvious that the disease was inflammation of the brain, and that the child was in great danger. Several leeches were applied to the mastoid process, a blister was put on the back of the neck, mercurial ointment was rubbed on the submaxillary glands, and two grains and a half of calomel were administered daily. On the seventh day of the treatment, the submaxillary glands were considerably swollen, the angle of the mouth was sore, and its cavity reddened, but there was no remarkable mercurial odour, and no commencement of salivation. On this day, 15 grains of calomel having now been taken, and two drachms of mercurial ointment rubbed on, a happy change took place, and gave great probability of a favorable termination. In the evening, the left cavity of the nose again secreted mucus, and afterwards the right cavity; the eyes (of which the left had been affected with œdema of the upper lid) again poured out tears when the child cried; and each external auditory meatus, but the left one more than the right, likewise began to secrete mucus. The return of these natural secretions, which deserve the greatest consideration, afforded the surest sign of the decrease of the morbid processes above mentioned. In order to aid these critical endeavours of nature, and give the last blow to the disease, Dr. Bierbaum used six more grains of calomel, and another drachm of mercurial ointment; so that, in all, twenty-one grains of calomel, and three drachms of mercurial ointment, had been employed. It was now time to discontinue the treatment, as was shown by the mercurial fever which came on, and which disappeared again in a few days. In this manner the child fortunately escaped its threatened fate; it recovered rapidly, and still enjoys the most perfect health.—*Ib., from Med. Zeit.*

---

*Dissection of case after the operation for Strabismus, by* PRESCOTT G. HEWETT, M. D.—George Clarke, æt 30, was admitted into St. George's Hospital, under the care of Mr. Babington, on November 11, 1840, for an ulcer of the leg. He was also affected with strabismus of the left eye, which was drawn outwards, the deviation being very considerable.

On the 1st December, Mr. Babington divided the left external rectus. Rather more inflammation than usual followed, but this subsided in a few days without any particular treatment. At the time of the operation the success appeared to be complete, but after the subsidence of the subsequent inflammation, it was evident that the defect, though much diminished, was not entirely removed, the axes of the two eyes being not absolutely in the same direction. The difference, however, was very slight, and it gradually lessened, so as to be scarcely perceptible, when the patient was attacked with inflammation of the lungs, supervening on tubercles, and died on January 1st, 1841.

The eye and its appendages were removed, and carefully dissected. It was found that the external rectus had been completely divided, just at the part where it was beginning to be tendinous; that the muscle itself had retracted to the distance of about three quarters of an inch from its natural attachment, but that it still remained connected with the globe by a strong band of cellular tissue. This band was about three lines in width, and about six lines in length, and was attached to the ball of the eye about two lines behind the original insertion of the muscle, and such was its strength that it allowed of being pretty forcibly pulled upon without giving way. *Med. Gaz.*

